# Risk of primary sclerosing cholangitis among patients with gastritis: a nationwide cohort study

**DOI:** 10.1007/s10654-025-01293-y

**Published:** 2025-08-23

**Authors:** Lina Lindström Älgå, Isabella Ekheden, Marcus Thuresson, Jonas F. Ludvigsson

**Affiliations:** 1 Department of Medicine Huddinge MedH, Karolinska Institutet, Stockholm, Sweden; 2 Division of Clinical Pharmacology, Department of Laboratory Medicine, Karolinska Institutet, Stockholm, Sweden; 3 Clinical Pharmacology, Karolinska University Hospital, Stockholm, Sweden; 4 Statisticon AB, Uppsala, Sweden; 5 Department of Medical Epidemiology and Biostatistics, Karolinska Institutet, Stockholm, Sweden; 6 Department of Pediatrics, Örebro University Hospital, Örebro, Sweden; 7 Department of Medicine, Columbia University College of Physicians and Surgeons, New York, NY USA

**Keywords:** Primary sclerosing cholangitis, Gastritis, *Helicobacter pylori*, Epidemiology

## Abstract

The pathogenesis of primary sclerosing cholangitis (PSC), a severe autoimmune liver disease, remains largely unknown. Infection with *Helicobacter pylori* (*H. pylori*) and subsequent gastritis could act as a triggering event of PSC, as *H. pylori* seems to be more prevalent in chronic liver disease. However, the risk of PSC among patients with gastritis and its precursor, *H. pylori* infection, is undetermined. In this nationwide cohort study, we included Swedish individuals undergoing a gastroscopy with biopsy during 1990–2017 showing gastritis (*n* = 306 588) or *H. pylori* (*n* = 11 890). Three control groups were used (1) matched controls from the Swedish general population (*n* = 1 544 667), (2) individuals with a gastric biopsy indicating normal mucosa (*n* = 318 754) and (3) sibling controls (*n* = 231 879). We calculated the hazard ratios (HRs) for PSC development, adjusting for age, sex, calendar year, county, comorbidities, alcohol-related disorders, education, and country of birth. Patients with a histological diagnosis of gastritis or *H. pylori* were more likely to be diagnosed with PSC during follow up. Compared to the general Swedish population, the fully adjusted HR for PSC among patients with gastritis was 3.35 (95% CI 2.67–4.20). However, compared to secondary controls with a normal gastric mucosa, the PSC risk was not increased among patients with gastritis. Patients with a gastroscopy biopsy showing gastritis have a moderately increased risk for PSC later in life but not compared to other individuals undergoing gastrointestinal work up (“normal mucosa)”. The association with PSC may be non-specific and apply to several gastrointestinal disorders.

**Supplementary Information:**

The online version contains supplementary material available at 10.1007/s10654-025-01293-y.

## Introduction

Primary sclerosing cholangitis (PSC) is a rare progressive liver disease, in most cases leading to recurrent cholangitis, liver cirrhosis and liver failure. Serious complications of PSC also include development of hepatobiliary cancers, in particular cholangiocarcinoma. PSC has a prevalence of 16/100 000 individuals in Sweden [1]. The disease is more common in males and about 80% of the patients have a concomitant inflammatory bowel disease (IBD). PSC remains an important indication for liver transplantation in Scandinavia [2].

The pathogenesis of PSC is largely unknown, and the disease has for long puzzled clinicians and researchers. PSC is believed to be an autoimmune disease most probably triggered by some event [3]. The initiating factors for the fibroinflammatory process in PSC remain obscure. Recent literature suggests that bacteria could act as a triggering antigen in PSC pathogenesis [4, 5] and small clinical studies have shown effect on liver function tests after treatment with antibiotics [6]. Siblings of patients with PSC and IBD are known to have a greater risk of developing PSC indicating that genetic factors are likely involved [7]. The lack of understanding the pathogenesis prevents development of therapy. Of note, a few studies have demonstrated an increased presence of *Helicobacter pylori* (*H. pylori*) in liver tissue with PSC [8, 9].

*Helicobacter pylori* is known to be the main cause of gastritis and is a major risk factor for gastric cancer [10]. The Correa cascade is a stepwise cascade towards gastric cancer where *H. pylori* infection is the first step [11]. The association of *H. pylori* with gastritis, peptic ulcer, dysplasia, neoplasia, and mucosa associated lymphoid tissue lymphoma (MALT) lymphoma, is well established. Furthermore, *H. pylori* has also been associated with several extra-gastric manifestations such as hepatobiliary and pancreatic diseases, other reports have also tried to link *H. pylori* infection to the development of autoimmune or immune mediated diseases, although results are diverging [12]. In IBD, a negative relationship between *H. pylori* and development of IBD has been described in epidemiological studies [13].

Here, we aimed to use a Swedish nationwide cohort to determine if exposure to gastritis or *H. pylori* is associated with PSC.

## Materials and methods

In this nationwide cohort study, we compared patients with a biopsy from the stomach or the duodenum showing gastritis, *H. pylori* or ulcer disease with three types of controls to determine if gastritis or *H. pylori* was associated with a later diagnosis of PSC.

### Data source

Several nationwide health data registers were linked to a histopathology-based cohort in Sweden, forming the ESPRESSO study, Epidemiology Strengthened by histopathology Reports in Sweden. The ESPRESSO study consists of gastrointestinal (GI) pathology reports collected at Sweden’s 28 pathology departments between 1965 and 2017 [14]. The cohort was assembled in 2015–2017 by collecting all Swedish histopathology data from the GI tract accompanied by complete information on date of biopsy, topography, and morphology. The cohort includes 2.1 million unique individuals and in total 6.1 million histopathology data entries (there are multiple biopsies in some individuals). Individuals from the histopathology cohort are matched with up to five controls from the general population as well as all first-degree relatives and first spouses to form a total study population of 13.0 million persons.

### Inclusion and exclusion criteria

**Fig. 1:**
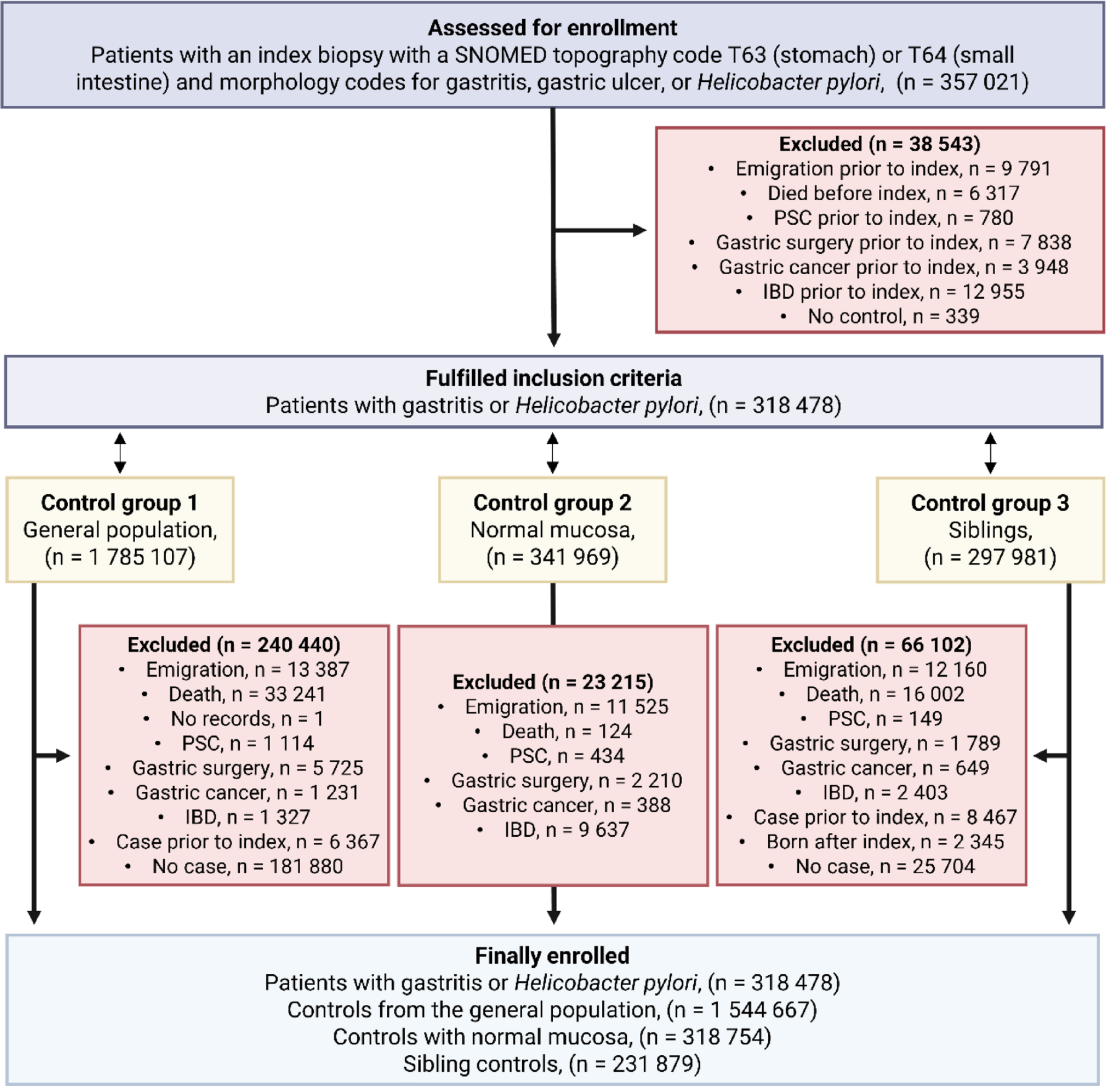
Flow-chart of the selection of study participants

Patients from the ESPRESSO study were included in the current study if they had a biopsy between 1990 and 2017 from the stomach or small intestine. The ESPRESSO study was restricted to individuals with a record of histopathology. We started the study period in 1990 since *H. pylori* was recently recognized and therefore poorly coded prior to that year. Exclusion criteria were if the patient prior to the first biopsy had either: (1) diagnosis of PSC, (2) diagnosis of IBD (required one ICD code and one relevant SNOMED histopathology code), (3) resection or surgical removal of the stomach or small intestine, (4) diagnosis of cancer in the stomach or small intestine (Fig. 1).

### Definition of cases and outcome

We identified all individuals undergoing a gastroscopy with biopsy from the stomach and or small intestine from the ESPRESSO cohort between 1990 and 2017. We used the following codes to identify exposed individuals: SNOMED topography codes T63 (stomach) and T64 (small intestine) and morphology codes M4X for gastritis, M38X ulcer disease and E13700, ME1370, E137, E1370 or E13701 for *H. pylori*.

Studying PSC from health registry data has not been frequently done due to the lack of a specific ICD code for PSC in earlier ICD versions [15, 16]. We therefore defined PSC as ever having an ICD code for cholangitis (ICD-7 585.29, ICD-8 575.05, ICD-9 576B or ICD-10 K83.0) in the Swedish Patient Register and at least one record of IBD (Crohn’s disease + Ulcerative colitis + IBD-U) and one SNOMED topography code according to our prior definition [17]. The date of PSC diagnosis was the first date when a patient had both an ICD code for cholangitis and fulfilled the criteria for IBD, whichever came first. PSC patients without IBD were therefore not included. The Patient Register has a positive predictive value of 85–95% for most diagnoses [18]. Our definition of IBD has shown a positive predictive value of 95% [19].

### Definition of controls

Three types of controls were used: (1) Up to five individuals from the general Swedish population, matched with cases at the date of index biopsy based on age, sex, calendar year (year of biopsy), and county, (2) patients from the ESPRESSO study with normal gastric mucosa (SNOMED codes: M00100 or M00110) and (3) full siblings identified through the Multi-Generation register. Exclusion criteria were also applied to controls.

### Statistical analysis

Patients with gastritis, gastric ulcer, or *H. pylori* in the study cohort were followed from the date of the first biopsy until the primary outcome (PSC) or death, emigration, eradication therapy for *H. pylori*, surgical removal of the stomach or small intestine or last date of follow-up 31st December 2019, whichever came first.

HRs for PSC among patients with gastritis, gastric ulcer, and *H. pylori* versus matched controls were determined using stratified Cox proportional hazards regression models, where the matching id (age, sex, calendar year and county) was the stratification factor. The initial model included group (case vs. control) and stratification factor. The adjusted models were further adjusted for (1) comorbidities (in the preceding 5 years), (2) alcohol-related disorders and diseases [20] (in the preceding 5 years), (3) education (≤ 9 years, 10–12 years, ≥ 13 years) and (4) country of birth (Nordic, not Nordic). Data on country of birth were retrieved from the Total Population Register [21] and data on education from the LISA database [22]. Comparisons vs. individuals with normal mucosa and vs. siblings were adjusted for age and sex in the initial model, and fully adjusted for the same variables as in the comparison vs. controls in the adjusted models.

Data were analyzed using R version 4.3.1. A *p* value < 0.05 was considered statistically significant.

### Ethics

This study was approved by the Stockholm Ethics Review Board. Individual informed consent was waived as the study was register-based [23].

## Results

### Background data

The median age at first biopsy with gastritis/*H. pylori* was 62 (IQR 46–74) years, and 55% of individuals were females (Table 1). The majority (84%) were born in the Nordic countries and 18% had college or university education. At baseline, the prevalence of diabetes, cancer, chronic obstructive pulmonary disease, obesity, or alcohol related disease in patients with gastritis/*H. pylori* was 7.6%, 8.5%, 2.6%, 3.8% and 2.1% respectively. Matched controls were similar regarding age, sex, and level of education (Table 1), but had less diabetes, cancer, chronic obstructive pulmonary disease, obesity, or alcohol related disease (3.6%, 4.5%, 1.3%, 2.0% and 0.9% respectively).

Individuals with a biopsy showing normal gastric mucosa had a substantially higher prevalence of diabetes, obesity, and cancer (4.7%, 2.9%, and 5.4% respectively) than controls, suggesting a higher degree of comorbidity than controls, despite being younger at baseline (median age: 44 (27–61) (Table 1). Siblings had less comorbidities compared to both matched controls and patients with normal mucosa biopsies (Table 1).

**Table 1:** Characteristics of study participants according to histological diagnosis of gastritis or *H. pylori*, compared with matched controls, normal mucosa controls and sibling controls

	Gastritis or *H. pylori* *N* = 357,022	Matched controls *N* = 1,785,108	Normal mucosa controls *N* = 341,969	Sibling controls *N* = 297,981
Sex				
Male, N (%)	143,680 (45)	695,253 (45)	118,648 (37)	117,437 (51)
Age				
Mean (SD)	58.2 (20.1)	57.8 (20.2)	44.4 (20.8)	46.4 (17.8)
Country of birth, N (%)				
Nordic	267,505 (84)	1,403,337 (91)	286,547 (90)	219,976 (95)
Other	50,973 (16)	141,330 (9)	32,207 (10)	11,903 (5)
Level of education, N (%)				
Compulsory school, ≤ 9 years	125,301 (39)	555,839 (36)	80,879 (25)	69,140 (30)
Upper secondary school (10–12 years)	110,779 (35)	539,020 (35)	128,873 (40)	98,332 (42)
College or university (≥ 13 years)	57,016 (18)	331,606 (21)	83,950 (26)	46,689 (20)
Missing	25,382 (8)	118,202 (8)	25,052 (8)	17,718 (8)
Year of diagnosis, N (%)				
1990–1999	120,712 (38)	589,728 (38)	89,217 (28)	70,433 (30)
2000–2009	127,736 (40)	617,717 (40)	139,610 (44)	97,938 (42)
2010–2016	70,030 (22)	337,222 (22)	89,927 (28)	63,508 (27)
Prevalence of comorbidity, N (%)				
Cancer	27,100 (8.5)	69,040 (4.5)	17,106 (5.4)	7503 (3.2)
Diabetes	24,148 (7.6)	55,858 (3.6)	14,947 (4.7)	6253 (2.7)
Chronic obstructive pulmonary disease (COPD)	8276 (2.6)	19,429 (1.3)	4508 (1.4)	1860 (0.8)
Obesity/dyslipidemia	12,197 (3.8)	30,336 (2.0)	9325 (2.9)	5541 (2.4)
Alcohol related disease	6700 (2.1)	13,315 (0.9)	4717 (1.5)	2750 (1.2)

### Main results

During a mean follow-up of 14 years, 144 patients with gastritis/*H. pylori* developed PSC (incidence rate: 3.7/100,000 person-years), compared to 240 events in matched controls (incidence rate: 1.2 /100,000 person-years) (Table 2). The corresponding fully adjusted HR was 3.35 (95% CI 2.67–4.20) (Table 2). The stratified HR was 3.17 (95% CI 2.55–3.94) (Table 2).

**Table 2:** Incidence rate and HRs (adjusted and unadjusted) of primary sclerosing cholangitis in individuals with gastritis or *H. pylori* compared with matched controls and normal mucosa controls

	Gastritis or *H. pylori* *N* = 318,478	Matched controls *N* = 1,544,667	Normal mucosa *N* = 318,754
Later diagnosis of PSC, N (%)	144 (0.045%)	240 (0.016%)	301 (0.094%)
Follow-up, years			
Median (IQR)	11.4 (6.2–18.3)	12.4 (7.2–19.0)	13.2 (8.1–19.6)
Incidence rate/100,000 PY (95% CI)	3.7 (3.1–4.3)	1.2 (1.0-1.3)	6.8 (6.0-7.6)
Hazard ratio (95% CI)			
Unadjusted	3.15 (2.56–3.87)/0.54 (0.44–0.66)	Ref. 1	Ref. 2
Stratified* or age- and sex-adjusted**	3.17 (2.55–3.94)/0.64 (0.52–0.79)	Ref. 1	Ref. 2
Fully adjusted***	3.35 (2.67–4.20)/0.70 (0.56–0.86)	Ref. 1	Ref. 2

*Stratified on matching id when comparing gastritis/*H. pylori* vs. matched controls

**Age- and sex-adjusted when comparing gastritis/*H. pylori* vs. normal mucosa

****Fully adjusted for comorbidities (in the preceding 5 years) + alcohol-related disorders and diseases (in the preceding 5 years) + education (≤ 9 years, 10–12 years, ≥ 13 years) + country of birth (Nordic, not Nordic)

**Fig. 2:**
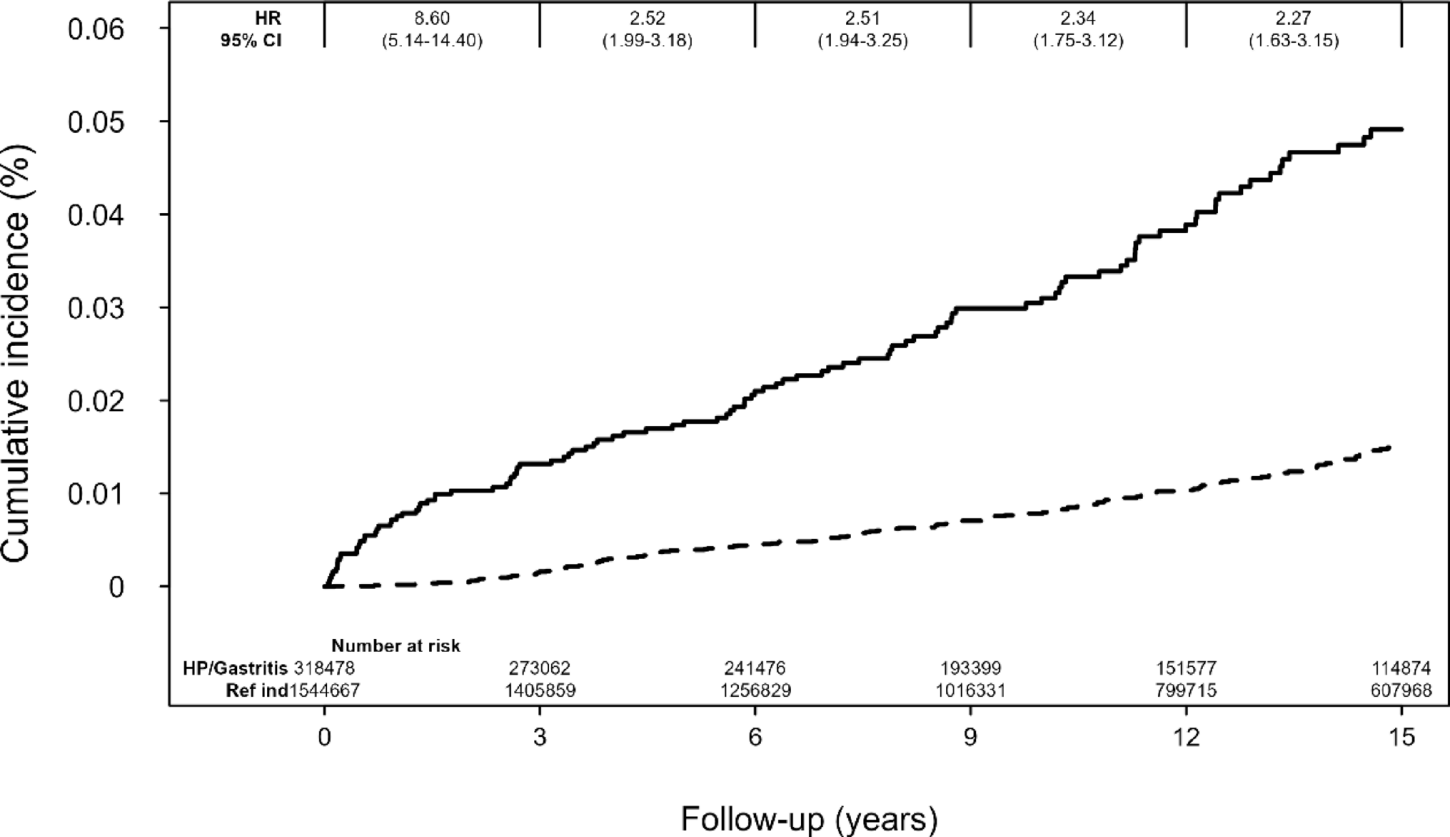
Kaplan–Meier curve of the cumulative incidence of PSC after a diagnosis of gastritis or *H. pylori*

The association between a diagnosis of gastritis/*H. pylori* was stable during the follow up period, as shown by the cumulative incidence curves in Fig. 2.

When looking separately at the individuals with *H. pylori* infection (*n* = 11,890), the number of PSC cases was very low, in total 7 patients. The HR for developing PSC followed a similar trend as compared with gastritis with an adjusted HR of 3.45 (95% CI 0.97–12.34) although the result was not statistically significant, due to inadequate power.

### Secondary controls (individuals with normal mucosa)

Compared to individuals with normal gastric mucosa, individuals with gastritis/*H. pylori* had a *lower* risk of future PSC (adjusted HR of 0.70 (95% CI 0.56–0.86)). During a median follow up of 13 years, 301 of the individuals with normal mucosa developed PSC, corresponding to an incidence rate per 100 000 person-years of 6.8 (95% CI 6.0–7.6). Individuals with gastritis/*H. pylori* remained at a lower risk of PSC also after adjustment for age- and sex (0.64; 95% CI 0.52–0.79) (Table 2).

### Sibling controls

Adjusting (stratified by family identity and fully adjusted) for confounders, patients with gastritis/*H. pylori* were at a higher risk of developing PSC than their siblings (HR = 2.94; 95% CI 2.00–4.33) (Table 3).

### Sensitivity analysis

As a sensitivity analysis we performed our main analysis starting follow-up 1 year after biopsy. In this analysis, few cases of PSC were diagnosed within 365 days after the initial biopsy and similar risks for developing PSC were found (Supplementary Table 1).

**Table 3:** Cox-regression analysis comparing risk of PSC in subjects with gastritis or *H. pylori* vs. their siblings

	Gastritis/*H. pylori*	Sibling
N	318,478	231,879
Events, n(%)	144 (0.045%)	72 (0.031%)
Follow-up years		
Mean (sd)	12.3 (8.0)	15.8 (7.5)
Median (IQR)	11.4 (6.2–18.3)	15.3 (9.6–21.7)
Incidence rate/100,000 PY (95% CI)	3.7 (3.1–4.3)	2.0 (1.5–2.5)
Hazard ratio (95% CI)		
Unadjusted	1.93 (1.45–2.56)	
Stratified*	2.74 (1.91–3.95)	
Fully adjusted**	2.94 (2.00–4.33)	

*Stratified by family identity + adjusted for age and sex

**Fully adjusted for comorbidities (in the preceding 5 years) + alcohol-related disorders and diseases (in the preceding 5 years) + education (≤ 9 years, 10–12 years, ≥ 13 years) + country of birth (Nordic, not Nordic)

## Discussion

In this nationwide cohort study in Sweden, we found that individuals with a previous histological diagnosis of gastritis or *H. pylori* carried an increased risk of developing PSC compared with matched controls from the general population. The fully adjusted HR for PSC among patients with gastritis/*H. pylori* was 3.35 (95% CI 2.67–4.20) compared to the background population and 2.94 (95% CI 2.00–4.33) compared to their siblings. Nonetheless, the increased risk disappeared when we compared with individuals with a histologically normal mucosa (in fact, the HR was < 1). These findings may at first seem contradictory but could have several explanations. Although a link between *H. pylori* and PSC has been postulated [24] and a significant association between *H. pylori* and PSC has been reported in a histological analysis of liver biopsies [8], our findings suggest other than causal explanations for this link.

The non-specific increase may be due to low-grade inflammation that may not have been detected at histological examination. It could also be that a high proportion of patients undergoing GI investigation have an impaired mucosal barrier, a different microbiota or other immune dysregulation (PSC has been linked to autoimmunity, which does not always show in the intestinal mucosa). Finally, we cannot rule out a shared genetic predisposition for several GI disorders and PSC or residual confounding including different patterns of healthcare seeking behavior.

Some individuals undergoing endoscopy with biopsy (showing either gastritis or normal mucosa), may have undergone GI investigations because of pre-diagnostic PSC symptoms such as upper abdominal pain. That some PSC diagnoses were made just after start of follow-up could potentially bias our results. However early PSC diagnoses were rare and hence unlikely to explain our findings (only 15% of all PSC was diagnosed during the first year of follow-up).It is also possible that individuals undergoing a biopsy (independent of the result) have more frequent healthcare visits and have an increased risk of surveillance and therefore are diagnosed with PSC more often and at an earlier stage. PSC that might not have been diagnosed in a control population where physician visits are fewer.

The diagnosis of PSC in this study was based on a combination of ICD codes for PSC and IBD. Identifying PSC cases solely through ICD coding (without a concomitant IBD code) remains challenging, and, unfortunately, there is no completely reliable method to ascertain true cases without manual review of individual records (which was beyond the scope of this article), and applying PSC-only ICD codes could lead to false-positive cases. In a previous nationwide registry-based study of PSC, the authors initially did not restrict their cohort to patients with concomitant IBD and identified an unexpectedly large cohort of 15,094 patients with cholangitis (ICD code K83.0) [16].

The diagnosis of *H. pylori* was in this study based on histology. Here, there is no information on why patients were investigated or why a biopsy was performed. Histology is probably not the most common diagnostic method for *H. pylori*. Treatment standards in patients with dyspeptic symptoms in primary care have in Sweden included the so called “Hp-test and treat” strategy, where Hp-positive dyspeptic patients are tested with a non-invasive, indirect Hp-test method [urea-breath-test (UBT), fecal ELISA-test for Hp-antigen (F-Hp or serology)] and treated with proton pump inhibitors and antibiotics without undergoing gastroscopy [25], provided they were younger than 50 years and had no alarm symptoms (bleeding, anemia, positive fecal hemoglobin test, weight loss, dysphagia, vomiting, abdominal mass) [26]. Some cases of gastritis and *H. pylori* were therefore not included in this study, since a proportion of *H. pylori* cases were probably diagnosed through rapid urease tests (i.e. CLO-test) alone. This may explain why our study was underpowered to detect a statistically significant increase for PSC among patients with *H. pylori* infection in the absence of gastritis. As a sensitivity analysis we stratified patients depending on the type of gastritis. Unfortunately, we did not have sufficient statistical power to examine the association of PSC with autoimmune gastritis or intestinal metaplasia. Furthermore, autoimmune gastritis typically defined as “chronic atrophic gastritis”, has often an *H. pylori*-dependent etiology rather than an autoimmune origin (and was hence not examined in this study).

As shown in Table 1, patients with both gastritis/*H. pylori* and normal mucosa had substantially more comorbidities than the controls. Although adjustments were made for comorbidities, alcohol-related disorders and diseases, education (≤ 9 years, 10–12 years, ≥ 13 years) and country of birth (Nordic, not Nordic) we cannot rule out that underlying comorbidity in the two biopsied groups has contributed to the excess PSC risk. Also, the two groups may have slightly different health-seeking patterns than the general population, strengthening any association with PSC. Although adjustments were made for comorbidities, residual confounding cannot be completely ruled out.

It has been hypothesized that *H. pylori* infection may play a role in the pathogenesis of autoimmune liver diseases, including PSC. Proposed mechanisms include molecular mimicry, whereby bacterial antigens share structural similarities with host biliary or hepatic proteins, potentially leading to aberrant immune activation [27].

Given that the ESPRESSO cohort is based on histopathology samples we did not have data on gastritis without biopsy. Hence, we cannot rule out that mild gastritis not requiring endoscopy has a different relationship with PSC. On the other hand, normal mucosa was also associated with PSC, arguing against that any gradient in gastritis severity would explain our findings.

As for the classification of patients with *H. pylori* in the ESPRESSO study, we could only capture cases with an *H. pylori* infection that was verified by a direct test on a gastroscopy-obtained biopsy at the pathology department (such as rapid urea test at biopsy or histology). Since the ESPRESSO study does not contain information on negative tests results (such as negative *H. pylori* tests), we assumed that most general population controls were *H. pylori* negative but cannot rule out some differential misclassification. Nevertheless, this potential differential misclassification is expected to attenuate our results and if *H. pylori* positive controls were excluded, this would most likely strengthen the association found in this study.

Additionally, we cannot rule out that there are other PSC risk factors shared by individuals with gastritis/*H. pylori* and those with normal mucosa such as exposure to certain medications, unmeasured comorbidity, similar health-care seeking behavior or other residual confounding.

In conclusion, our data suggest that patients with gastritis/*H. pylori* are at increased risk of PSC, but it seems unlikely that this risk is exclusively due to the degree of inflammation or infection. Consequently, we also conclude that it is unlikely that treatment of gastritis/*H. pylori* will reduce the future risk of PSC as the PSC risk was not increased compared to patients with a normal mucosa.

## Supplementary Information

Below is the link to the electronic supplementary material.

Supplementary file1 (DOCX 14 KB)

## Abbreviations

GI: Gastrointestinal
HP: Helicobacter pylori
HR: Hazard ratio
IBD: Inflammatory bowel disease
ICD: International Classification of Diseases
PSC: Primary sclerosing cholangitis
PY: Person years
CLO: Campylobacter like organism test
