# Supplementary material for: Risk of primary sclerosing cholangitis among patients with gastritis: a nationwide cohort study
Source: Eur J Epidemiol. 2025 Aug 23;40(10):1243–50. doi: 10.1007/s10654-025-01293-y (PMC12660386; doi:10.1007/s10654-025-01293-y)
Supplement: Supplementary file 1 — Supplementary file1 (DOCX 14 KB) [file 10654_2025_1293_MOESM1_ESM.docx]

| **Supplementary table 1.** Cox regression analysis of PSC starting follow-up after 1 year in individuals with gastritis or *H. pylori* compared with matched controls and normal mucosa controls. Hazard ratios (95% CI) first vs Matched controls/Normal mucosa | | | |
| --- | --- | --- | --- |
|  | **Gastritis or *H. pylori***  **N = 294 338** | **Matched controls**  **N = 1 496 737** | **Normal mucosa**  **N = 306 528** |
| **Events, n (%)** | 122 (0.041%) | 237 (0.016%) | 267 (0.087%) |
|  |  |  |  |
| **Follow-up years** |  |  |  |
| **Mean (sd)** | 12.3 (7.5) | 12.7 (7.5) | 13.5 (7.3) |
| **Median (IQR)** | 11.3 (6.2–17.9) | 11.8 (6.7–18.2) | 12.7 (7.7–18.9) |
|  |  |  |  |
| **Incidence rate/100000 PY (95% CI)** | 3.4 (2.8-4.0) | 1.2 (1.1–1.4) | 6.5 (5.7–7.3) |
|  |  |  |  |
| **Hazard ratio (95% CI)** |  |  |  |
| **Unadjusted** | 2.71 (2.18–3.37)/  0.52 (0.42–0.65) | Ref. 1 | Ref. 2 |
| **Stratified** | 2.69 (2.14–3.39)/  0.62 (0.49–0.77) | Ref. 1 | Ref. 2 |
| **Adjusted** | 2.89 (2.28–3.67)/  0.66 (0.53–0.83) | Ref. 1 | Ref. 2 |
